# Retraction Note: Role of inhibitors of serine peptidases in protecting *Leishmania donovani* against the hydrolytic peptidases of sand fly midgut

**DOI:** 10.1186/s13071-023-05744-x

**Published:** 2023-03-27

**Authors:** Sudha Verma, Sushmita Das, Abhishek Mandal, Md Yousuf Ansari, Sujata Kumari, Rani Mansuri, Ajay Kumar, Ruby Singh, Savita Saini, Kumar Abhishek, Vijay Kumar, Ganesh Chandra Sahoo, Pradeep Das

**Affiliations:** 1 Department of Molecular Biology, Rajendra Memorial Research Institute of Medical Sciences (ICMR), Agamkuan, Patna, Bihar 800007 India; 2 Department of Microbiology, All India Institute of Medical Sciences, Patna, Bihar 801105 India; 3 National Institute of Pharmaceutical Education and Research, Hajipur, Bihar 844101 India; 4 MM College of Pharmacy, Maharishi Markandeshwar University, Mullana, Ambala 133207 India; 5 Department of Vector Biology, Rajendra Memorial Research Institute of Medical Sciences, (ICMR), Agamkuan, Patna, Bihar 800007 India; 6 Department of Bioinformatics, Rajendra Memorial Research Institute of Medical Sciences (ICMR), Agamkuan, Patna, Bihar 800007 India

**Retraction** **:** **Parasites & Vectors (2017) 10:303** 10.1186/s13071-017-2239-9

The Editor-in-Chief has retracted this article. After publication, concerns were raised regarding western blot similarities in Figs. 5B (α-tubulin, between lanes 1–3 and 5–7) and 6C (α-tubulin, between lanes 1–3 and 4–6). Additional checks by the Publisher have found a number of unexpected breaks in the western blot backgrounds in Figs. 3C–F and 5B.

The Editor-in-Chief therefore no longer has confidence in the presented data.

Sujata Kumari agrees to this retraction. Sudha Verma does not agree to this retraction. None of the other authors have responded to any correspondence from the editor or publisher about this retraction.

